# Medical management of endometriosis

**DOI:** 10.1097/GCO.0000000000000983

**Published:** 2024-08-17

**Authors:** Anais Alonso, Kate Gunther, Sarah Maheux-Lacroix, Jason Abbott

**Affiliations:** a School of Women's and Children's Health, University of New South Wales; b Gynaecological Research and Clinical Evaluation (GRACE) Unit, Royal Hospital for Women, Sydney, New South Wales, Australia; c Centre de recherche du CHU de Québec, Université Laval, Québec City, Québec, Canada

**Keywords:** complementary and alternative medicine, dyschezia, dysmenorrhea, dyspareunia, dysuria, endometriosis, medical management, pelvic pain, quality of life

## Abstract

**Purpose of review:**

While laparoscopic surgery plays a key role in the management of endometriosis, symptoms commonly recur, and repeat surgery comes with increased risk. Medical management, including hormonal and nonhormonal treatment, is vital in managing painful symptoms. This review summarizes recent evidence regarding various medical management options available to treat pelvic pain associated with endometriosis.

**Recent findings:**

Efficacy of dienogest vs. combined oral contraceptive on pain associated with endometriosis: randomized clinical trial.

Once daily oral relugolix combination therapy vs. placebo in patients with endometriosis-associated pain: two replicate phase 3, randomised, double-blind, studies (SPIRIT 1 and 2).

A randomized, double-blind, placebo-controlled pilot study of the comparative effects of dienogest and the combined oral contraceptive pill in women with endometriosis.

Two-year efficacy and safety of relugolix combination therapy in women with endometriosis-associated pain: SPIRIT open-label extension study.

**Summary:**

All symptomatic women with suspected endometriosis who are not desiring immediate fertility can be offered suppressive treatment to control symptoms and slow the progression of disease. First-line treatments include the combined oral contraceptive pill and progestogens. Second-line treatments include gonadotropin-releasing hormone agonists and antagonists but current guidelines recommend that these should be reserved for people whose symptoms fail to be controlled by first-line agents. The use of complementary and alternative medicines is also increasing in both volume and number of agents used.

## INTRODUCTION

Endometriosis is defined by the presence of endometrial-like tissue outside the uterine cavity. It affects 11.4% of reproductive-aged females [1] and commonly causes chronic pelvic pain and infertility. Laparoscopy remains the gold standard for diagnosis of small volume disease, with imaging recognized as being diagnostic in severe or invasive disease [2,3]. Surgical treatment of endometriosis is reported to improve pain up to five years postexcision of lesions [4]. However, women, girls and people from the trans and gender diverse communities (collectively referred to hereafter as “women”) face approximately six years between the onset of symptoms and diagnosis [5], and empirical treatment should be commenced early when there is clinical or radiological diagnosis of disease [2,3]. 

**Box 1:**
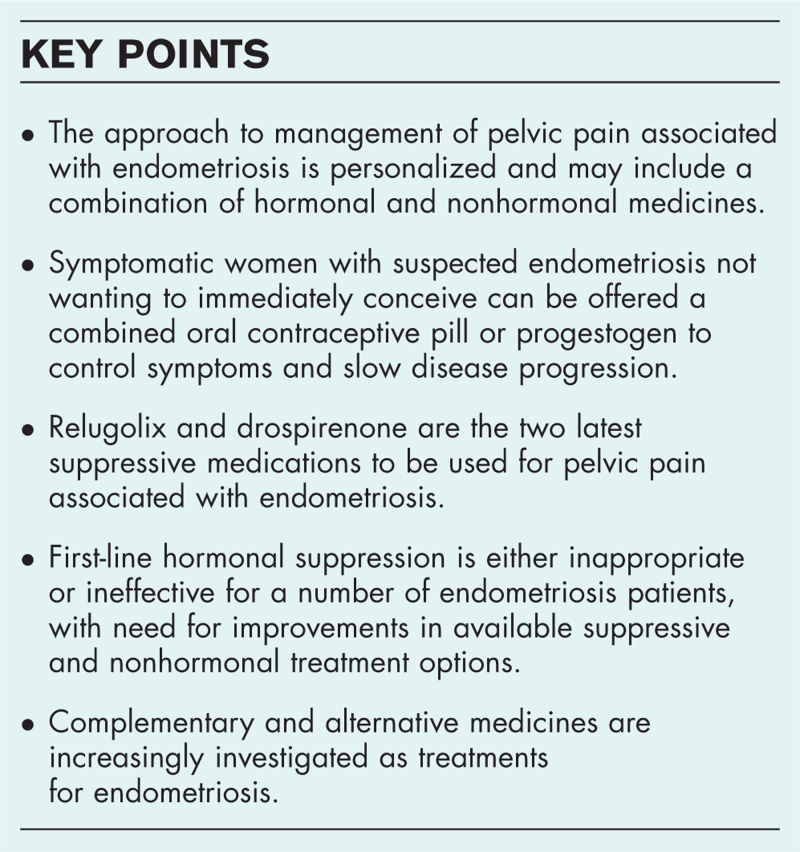
no caption available

**FIGURE 1:**
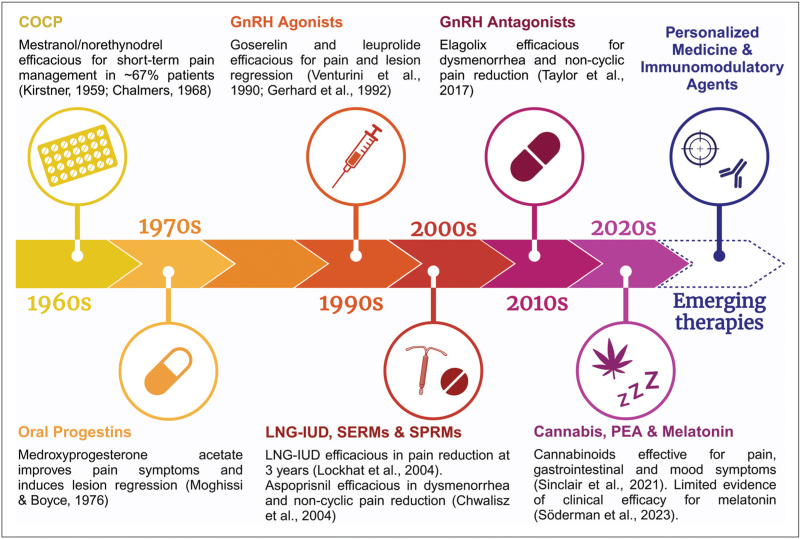
Timeline with key milestones in the historical development of treatments for endometriosis and emerging therapeutic approaches. Created with BioRender.com. COCP, combined oral contraceptive pill; GnRH, gonadotropin-releasing hormone; LNG-IUD, levonorgestrel intrauterine device; PEA, palmitoylethanolamide; SERM, selective estrogen receptor modulator; SPRM, selective progesterone receptor modulator.

Endometriosis has long been treated with hormonal therapies that impact estrogen. Figure 1 represents a timeline of these treatments over the past 70 years. First-line therapies include the combined oral contraceptive pill (COCP), progestogens and nonsteroidal anti-inflammatory drugs. Second-line suppressive treatments such as gonadotropin-releasing hormone agonists (GnRHa) and antagonists (GnRHant) can be offered to those whose symptoms fail to be controlled by first-line agents [3]. Nonhormonal complementary and alternative medicines are increasingly investigated as possible treatments for endometriosis.

The aim of this article is to review recent evidence for a number of medical options available to treat pelvic pain associated with endometriosis. In addition, a brief outline of medical management options for special populations is provided, as are areas of future research interest.

## METHODOLOGY

This literature review focuses on best quality evidence for medications that are either currently used as hormonal therapies or identified as published priority areas of research [6]. The scope of articles is contained to original research investigating pain and quality of life. We searched PubMed for relevant clinical trials published between December 2021 and March 2024 inclusive. The search strategy included ‘endometriosis’ AND (‘pain’ OR ‘dysmenorrhea’ OR ‘dyspareunia’ OR ‘dyschezia’ OR ‘dysuria’ OR ‘quality of life’) AND (‘oral contraceptive pill’ OR ‘progestin’ OR ‘levonorgestrel’ OR ‘gonadotropin releasing hormone’ OR ‘selective progesterone receptor modulator’ OR ‘selective estrogen receptor modulator’ OR ‘cannabis’ OR ‘melatonin’ OR ‘palmitoylethanolamide’). Abstracts were assessed for suitability. Where identified, the full text article in English was obtained and analyzed. References in included articles were hand searched for additional studies. In addition, clinical trial registries (Australian New Zealand Clinical Trials Registry, ClinicalTrials.gov, EU Clinical Trials Register, Health Canada's Clinical Trials Database, International Clinical Trials Registry Platform and International Standard Randomised Clinical Trial Number registry) were searched for randomized controlled trials (RCTs) for included complementary medicines.

## RESULTS AND DISCUSSION

### Hormonal medications

#### Combined oral contraception pill

International guidelines recommend offering suppressive treatment to all symptomatic women with suspected or confirmed endometriosis not wanting to immediately conceive, with the COCP and single-agent progestogens being first-line [2,3,7]. Despite its widespread use, prospective evidence for the COCP is limited to a handful of studies. A Cochrane meta-analysis of RCTs concluded that the COCP was associated with improvements in dysmenorrhea, dyspareunia and dyschezia compared to placebo [8].

There are few studies since, with a prospective observational study of 64 women with deep infiltrating endometriosis (DIE) receiving a COCP (2 mg dienogest and 30 μg ethinyl estradiol) in an extended regime finding the proportion of women suffering from severe dysmenorrhea and nonmenstrual pelvic pain (NMPP) significantly reduced after 2 years (dysmenorrhea: 82.5% baseline vs. 6.3% 12 months vs. 0% 24 months, *P* < 0.001; NMPP: 31.7% vs. 0% vs. 0%, *P* < 0.001). The mean pain score decreased for dysmenorrhea, NMPP, deep dyspareunia and dyschezia but not dysuria, likely due to very low baseline pain scores [9^▪^].

In another observational study, 42 women with DIE, 31 with DIE and adenomyosis and 39 controls (diagnosed via transvaginal ultrasound) were treated with the same COCP as above for 12 months. Treatment was associated with significant improvements in dysmenorrhea, NMPP, dyspareunia and dyschezia, quality of life and sexual quality of life for women with DIE, regardless of coexisting adenomyosis [10].

We are unlikely to see substantial improvements in available evidence for the COCP. Given its widespread use, there is little financial impetus, particularly for pharmaceutical companies, to invest in new high-quality studies.

### Oral progestins

Oral progestins have been used for over 50 years for managing endometriosis. More than 10 RCTs investigating pain symptoms are published, although most placebo-controlled RCTs are now dated, and meta-analysis comparing oral progestins with other suppressive therapies has not demonstrated superiority of one over the other [11].

This review found dienogest to be the most studied progestogen in recent years. In an RCT, 70 women with surgically or radiologically diagnosed endometriosis were randomized to receive dienogest 2 mg or a COCP (ethinyl estradiol 0.03 mg and drospirenone 3 mg) for 24 weeks. Both interventions significantly improved NMPP on the visual analogue scale (VAS) (mean difference: dienogest 6.0, 95% CI 4.9–7.1, *P* < 0.0001 vs. COCP 4.54, 95% CI 3.1–5.9, *P* < 0.0001), with dienogest noninferior to the COCP (mean change 1.42, 95% CI −0.33–3.18, *P* = 0.111). Notably, patients were excluded if they had previously failed to achieve adequate analgesia with the study drugs, suggesting patients with severe symptomatic or treatment-resistant endometriosis may not benefit. In addition, the dropout rate was high (29% in dienogest group vs. 26% in COCP group), mainly due to inadequate pain control or intolerable adverse effects, though a modified intention-to-treat analysis was performed [12].

In a separate double-blind RCT, 108 women were given dienogest 2 mg, a COCP (ethinyl estradiol 30 μg and levonorgestrel 0.3 mg) or placebo postlaparoscopic excision of stage IV endometriosis without bladder or bowel resection. After 6 months of treatment, both dienogest and the COCP improved overall pelvic pain via VAS (mean difference: dienogest −5.39 ± 3.81, *P* = 0.011 vs. COCP −5.79 ± 4.11, *P* = 0.010) but not placebo (−3.14 ± 0.66, *P* = 0.393). Again, no difference in treatment effect was observed between the intervention groups [13].

Drospirenone is the most recent single-agent progestogen to be studies for endometriosis, although evidence is limited to one retrospective analysis of 61 adolescents, with self-reported improvements in dysmenorrhea and pelvic pain in 46% and 62% of users respectively [14].

### Levonorgestrel intrauterine device

In a Cochrane systematic review examining the effects of the LNG-IUD for endometriosis-associated symptoms, dysmenorrhea and quality of life were improved compared to expectant management [15].

Original research from the data extraction period notes a retrospective study of long-term effectiveness of the LNG-IUD compared to a COCP (ethinyl estradiol 20 μg and drospirenone 3 mg) and dienogest 2 mg postoperatively [16]. The 1-year postoperative decrease in NMPP/back pain (4.0 ± 1.6 baseline vs. 0.6 ± 1.3 1-year, *P* < 0.001), dysmenorrhea (6.5 ± 1.7 vs. 0.8 ± 1.4, *P* < 0.001) and dyspareunia/dyschezia (4.1 ± 1.1 vs. 1.3 ± 1.4, *P* = 0.006) was sustained at up to 10 years postsurgery with continued LNG-IUD use. However, there is a substantive risk of attrition bias due to exclusion of those lost to follow up within the first 5 years. This likely includes women who discontinued the intervention due to intolerable side effects or inadequate analgesia.

**FIGURE 2:**
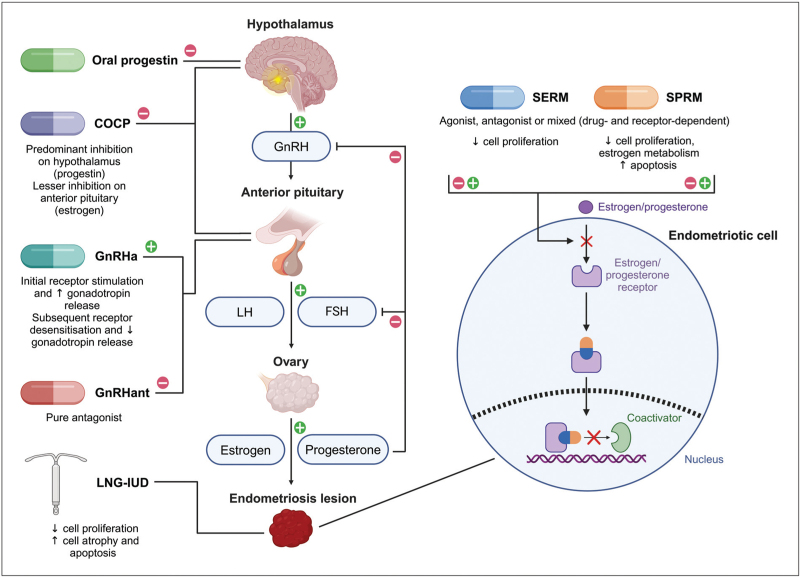
Mechanism of action of the suppressive medications discussed in this article. Gonadotropin-releasing hormone agonists and antagonists, the combined oral contraceptive pill and oral progestins act on the hypothalamic-pituitary-ovarian axis, while the levonorgestrel intrauterine device exerts local effects on endometriosis lesions. Selective estrogen and progesterone receptor modulators interact with their respective nuclear receptors within endometriotic cells. Adapted from “Hypothalamic-Pituitary-Ovarian Axis” and “Tamoxifen Signaling in Breast Cancer”, by BioRender.com (2024). Retrieved from: https://app.biorender.com/biorender-templates. COCP, combined oral contraceptive pill; FSH, follicle-stimulating hormone; GnRHa, gonadotropin-releasing hormone agonist; GnRHant, gonadotropin-releasing hormone antagonist; LH, luteinizing hormone; LNG-IUD, levonorgestrel intrauterine device; SERM, selective estrogen receptor modulator; SPRM, selective progesterone receptor modulator.

The choice of first-line hormonal agent should be driven by a need to avoid estrogen (e.g., for patients who have migraine with aura) or specific desirable effects. This may include ovulatory suppression where the COCP and some oral progestogens [17] are superior to the LNG-IUD. The compliance (when well tolerated) and convenience of the LNG-IUD is excellent and counselling should encompass both the medical effect desired and the patient's wishes. Figure 2 graphically represents the mechanism of action of the suppressive agents discussed in this section.

### Gonadotropin-releasing hormone agonists and antagonists

GnRHa have been used since the 1980s for management of endometriosis symptoms, however their role is limited due to their adverse effects profile, with particular concern for osteoporosis and heart disease. GnRHa should be reserved for those who have failed first-line suppressive treatments and administered short-term with the use of hormonal add-back [3].

GnRHant are the newest hormone suppressive variant to be investigated and marketed for endometriosis. Partial hypoestrogenism is induced in a dose-dependent manner, minimizing many side effects observed with GnRHa [18]. The replicate phase III multicenter SPIRIT 1 and 2 studies are double-blind, placebo-controlled RCTs comparing relugolix 40 mg and add-back (estradiol 1 mg and norethisterone acetate 0.5 mg) against relugolix monotherapy with delayed add-back from 12 weeks against placebo in 638 and 623 women with moderate-to-severe NMPP or dysmenorrhea and previously surgically diagnosed endometriosis [19]. Relugolix combination therapy was more effective than placebo in reducing both dysmenorrhea (SPIRIT 1: 75% response rate with relugolix combination vs. 72% delayed relugolix combination vs. 27% placebo, 47.6% treatment difference between relugolix combination and placebo, 95% CI 39.3–56.0, *P* < 0.0001; SPIRIT 2: 75% vs. 73% vs. 30%, 44.9% difference, 95% CI 36.2–53.5, *P* < 0.0001) and NMPP (SPIRIT 1: 59% vs. 58% vs. 40%, 18.9% difference, 95% CI 9.5–28.2, *P* < 0.0001; SPIRIT 2: 66% vs. 53% vs. 43%, 23.4% difference, 95% CI 13.9–32.8, *P* < 0.0001).

The same cohort was followed up in the SPIRIT open-label extension trial, with 501 participants completing a further 80 weeks of relugolix combination therapy [20^▪▪^]. Among those initially allocated to the relugolix combination group, the response rates for dysmenorrhea and NMPP were 84.8% and 75.8% respectively. Bone mineral density (BMD) decreased by 0.45%, 0.09% and 0.56% compared to baseline in those originally allocated to the relugolix combination, delayed combination and placebo groups respectively.

The GnRHant agents relugolix, linzagolix [21] and elagolix [22] provide the convenience of oral delivery with a seemingly improved side effect profile and lower risk compared with GnRHa. Comparative studies of GnRHant with first-line agents are required to better define their precise role in the clinical management of endometriosis that includes both clinical and cost-efficacy as they currently are a much more expensive option than many first-line treatments.

### Selective progesterone and estrogen receptor modulators

SPRMs have been studied for their effects on endometriosis-associated symptoms. A Cochrane review reported that mifepristone significantly improved dysmenorrhea compared to placebo, and that ulipristal acetate was equally efficacious as leuprolide in treating pelvic pain, with decreased severity and fewer days of pain [23]. There has been little new research into this drug class given prescription restrictions for ulipristal acetate following reports of serious liver injury [24] occasionally requiring transplantation [25], and concerns for drug toxicity in long-term murine trials of vilaprisan [26].

The SERM raloxifene, used for postmenopausal osteoporosis, was trialed for endometriosis-associated pain, however the RCT was terminated early due to increased pain and earlier repeat laparoscopy [27]. A novel SERM, SR-16234, has recently undergone one open-label single arm clinical trial [28]. Ten patients, nine with endometriosis and one with adenomyosis, were given SR-16234 40 mg daily for 12 weeks. Pelvic pain and dysmenorrhea were reported to be significantly improved, although no quantitative data are provided. Use of this drug class is currently limited to research protocols.

### Complementary and alternative medicines

The following medications are not approved for use in endometriosis by the Food and Drug Administration, and local guidance must be followed if considering their use.

### Cannabis

An international survey reports that 49% of people with endometriosis have used cannabis for symptom management [29], although selection bias and a lack of robust evidence exploring analgesic effects are noted. In a retrospective analysis of 246 patients with self-reported endometriosis, 42.4% used cannabis for pelvic pain, with gastrointestinal distress, cramps, nausea, depression and reduced libido reportedly improved [30]. Australian and New Zealand cross-sectional survey data suggest similarly promising results, with 45% and 39% of cannabis users with endometriosis reporting “much better” chronic pelvic pain and dysmenorrhea respectively [31]. In another cross-sectional survey, pain was reduced by 7.6 ± 2.0 on an 11-point scale for those using cannabis for symptom management. Fifty-six percent of women reported a reduction in pharmacotherapy use by >50%, predominantly opioids and benzodiazepines [32].

Lack of regulation and disparities in product potency make it difficult to conduct well controlled studies. Given the potential legal ramifications and side effects alongside increasingly widespread use, attainment of high-quality evidence is imperative. There are a number of registered placebo-controlled RCTs on cannabis use for endometriosis (ACTRN12622001560785, ACTRN12624000328572, NCT05670353, RBR-6ryrpjs).

### Melatonin

Melatonin was proposed as a therapeutic option for endometriosis after murine endometrium autograft studies observed a reduction in lesion volume following intraperitoneal injection [33,34]. A double-blind RCT of 40 women with surgically or radiologically diagnosed endometriosis and severe dysmenorrhea were given melatonin 20 mg or placebo before sleep. The authors found no difference in pain (adjusted mean via numerical rating scale: melatonin 2.9 ± 1.9 vs. placebo 3.3 ± 2.0, *P* = 0.446), dysuria (1.1 ± 1.8 vs. 1.1 ± 1.7, *P* = 0.930), dyschezia (1.0 ± 1.7 vs. 1.7 ± 2.0, *P* = 0.263) or dyspareunia (0.7 ± 1.2 vs. 1.1 ± 1.8, *P* = 0.499) after 2 months of treatment [35^▪^]. Notably, patients were recruited based on the presence of severe dysmenorrhea but this was not an outcome. Additionally, with a short half-life of only 20–40 min [36], it is unclear if analgesia can be achieved during waking hours.

### Palmitoylethanolamide

**FIGURE 3:**
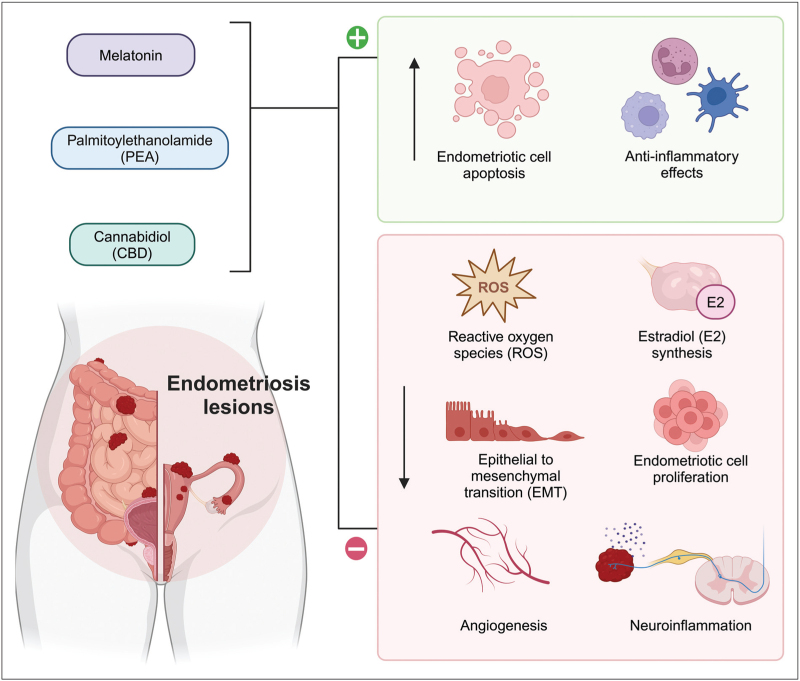
Mechanism of action of complementary and alternative medicines under investigation for endometriosis treatment. Cannabidiol (CBD), melatonin, and fatty acid amide palmitoylethanolamide (PEA) bind to membrane-bound receptors and act as agonists of peroxisome proliferator-activated receptor alpha (PPAR-α) in neurons and immune cells to exert systemic anti-inflammatory effects, reduce estradiol production and mediate afferent pain signals in the central nervous system. Within the endometriotic niche, these agents inhibit production of reactive oxygen species (ROS), reducing angiogenesis, cell proliferation, and epithelial to mesenchymal transition (EMT). Created with BioRender.com.

Palmitoylethanolamide (PEA) has been investigated for its anti-inflammatory and analgesic properties, with one 3-arm RCT recruiting 61 women with mild-to-moderate endometriosis who were given daily micronized PEA combined with transpolydatin or placebo for three months, or celecoxib twice daily for seven days after laparoscopy. PEA was more effective than placebo but inferior to celecoxib in treating dysmenorrhea, dyspareunia and pelvic pain (no quantitative data, *P* < 0.001) [37]. There is one placebo-controlled RCT registered to investigate PEA and endometriosis (ACTRN12620001311943). Figure 3 graphically represents the complementary and alternative treatment options discussed in this section.

## PERSONALIZED MEDICINE

### Primary prevention

Primary prevention of endometriosis could change the landscape of management yet remains elusive given the uncertainty surrounding disease etiology. Evidence suggests that current use of oral contraceptives protects against endometriosis however this benefit is not conferred to past users, which may suggest that the true effect is simply a delay in diagnosis due to symptomatic control [38]. Lifestyle interventions, including exercise, diet and alcohol intake have been studied but results remain unclear [3].

### Progesterone resistance

While hormonal suppression may be offered to all symptomatic women not wanting to immediately conceive, approximately one-third will not respond to first-line suppressive therapies due to progesterone resistance [39].

There is emerging evidence to suggest that endometriosis may exist as multiple molecular subtypes, with the identification of distinct stroma-enriched (S1) and immune-enriched (S2) lesions [40^▪▪^]. S1 lesions demonstrate fibroblast activity and extracellular matrix remodeling, while S2 lesions exhibit immune cell upregulation and infiltration, and are strongly associated with failed suppressive therapy. Further research may facilitate classification of distinct disease states, not merely to distinguish potential etiological differences, but to guide personalized management. This must also overcome the need for surgery to retrieve lesions for that diagnosis, as this invasive step may be of greater risk and cost than a failed trial of progestogen-containing medical treatment.

### Pregnancy

Women have long been told that pregnancy will alleviate their pain symptoms, however there is limited evidence to support this [41]. Suppressive medications are clearly contraindicated in pregnant women and safety of complementary therapies is unclear. Until more robust evidence becomes available, medical management during this period should be limited to nonhormonal analgesics.

### Post-menopause

Most women who experience endometriosis symptoms postmenopause use menopausal hormone therapy, which may be withdrawn if their pain is more troublesome than their menopausal symptoms. Due to the uncertainty of diagnosis and risk of malignancy with older age, surgical management often plays a greater role in this population. Clinicians can consider aromatase inhibitors for pain symptoms, though evidence is extremely limited with just six case studies suggesting an improvement in pain [42].

## FUTURE DIRECTIONS

Dysregulation of the innate and adaptive immune systems is an integral component of both the systemic and local endometriosis microenvironments, which may indicate a role for immunomodulatory therapeutics [43]. Recent murine studies have demonstrated positive results on autograft endometrium transplants with existing immunotherapies such as rituximab and stem cells [44,45], and novel gene and nanoparticle treatments [46,47], though publication bias is likely present. Additionally, preclinical efficacy, usually defined as lesion size reduction, remains inconclusive in its translation to clinical symptoms.

Evidence from human studies is limited to one prospective study in which 11 women were given either one or two doses of intravenous methotrexate. Improvements in deep dyspareunia and dyschezia were reported 180 days posttreatment. There was no significant difference in dysmenorrhea, dysuria or chronic pelvic pain [48^▪^]. Another clinical trial is currently recruiting patients for treatment with HMI-115, a monoclonal antibody, after promising preclinical murine data (NCT05101317) [49].

## CONCLUSION

Current evidence for most pharmacotherapies to treat pain symptoms associated with endometriosis is variable. Traditionally, there has been substantive focus on hormonal suppression, which is sufficient to achieve adequate symptom control in the majority of patients. However, for the proportion who still have persistent symptoms despite this, there is clear impetus to improve upon available medical treatment options.

## Acknowledgements

*None.*

### Financial support and sponsorship

*None.*

### Conflicts of interest

*A.A. holds shares in CSL and Ecofibre. S.M.L. has received grants or honoraria from Bayer, AbbVie, Ethicon and Pfizer. J.A. reports funding through the Medical Research Future Fund (Australian Government), Australasian Gynaecological Endoscopy and Surgery (AGES) Society and Endometriosis Australia for research programs. He is a Chairman of the National Endometriosis Clinical and Scientific Trials network (Government funded), and a member of the Endometriosis Advisory Group Member to the Australian government and Steering committee for the National Action Plan for Endometriosis. He is a member of the Internation Federation of Gynaecology and Obstetrics (FIGO) Committee on Menstrual Disorders and Related Health Impacts and Co Editor-in-Chief of the Journal of Minimally Invasive Gynecology. He is on the scientific advisory board for Hologic and Gedeon Richter and has been a consultant/speaker for Bayer, MSD, Stryker, Karl Storz, Allergan, Vifor, and Organon. K.G. has no conflicts of interest.*

## REFERENCES AND RECOMMENDED READING

Papers of particular interest, published within the annual period of review, have been highlighted as:

▪ of special interest
▪▪ of outstanding interest
